# Comparison of the methods for platelet rich plasma preparation in horses

**DOI:** 10.1186/s40781-018-0178-4

**Published:** 2018-08-18

**Authors:** Eun-bee Lee, Jung-Won Kim, Jong-pil Seo

**Affiliations:** College of Veterinary Medicine and Veterinary Medical Research Institute, Jeju National University, Jeju-City, Jejudo 63243 Republic of Korea

**Keywords:** Platelet rich plasma, Red blood cells, White blood cells, Horse

## Abstract

Platelet rich plasma (PRP) is popularly used in the horse industry to enhance regeneration of tissue injury that has limitation of blood supply. This study aimed to compare the methods for platelet rich plasma preparation since they has not been established yet. Blood was collected from six horses and platelets were concentrated by three different methods (2-step centrifugation, separated centrifugation and separated centrifugation using histopaque). Concentrated blood was analyzed using Advia hematology systems. In the result, separated centrifugation with histopaque showed the significantly lower number of red blood cells than other groups. The 2-step centrifugation showed the significantly higher number of white blood cells than other groups, while it contained the highest concentration of red blood cells among three groups. In the 2-step centrifugation, separated centrifugation and separated centrifugation with histopaque, platelets were concentrated 4.5, 5.3 and 5.6 times, respectively. And no significant difference of the platelet concentration between the three groups was found. This study demonstrated that separated centrifugation using histopaque was the best method for platelet rich plasma preparation because of the proper amount of platelets and the separation of red blood cells from platelet rich plasma.

## Introduction

Platelet rich plasma (PRP) is the blood plasma which contains a high concentration of platelets. Recently, PRP is largely used in the treatment of equine musculoskeletal, soft tissue, skin injuries [1, 2]. It includes α-granules that secrete the growth factors, including platelet-derived growth factor (PDGF), transforming growth factor beta (TGF-β) and vascular endothelial growth factor (VEGF) [3–5]. The growth factors help the migration of the cells and the regeneration of blood vessels, ligaments, tendons, bones and skin [1, 6]. In horses, there are 100,000–350,000 platelets/ul in the blood and platelet rich plasma should contain three times to five times of platelets [7]. However, there is no establishment of the methods for platelet rich plasma preparation [8]. Therefore, the purpose of this study is a comparison of the three different methods for platelet rich plasma preparation.

## Materials and methods

Six horses were used in this study (two of Thoroughbred and four of Halla horses; two males and four females; mean age, 8 years; mean weight, 340 kg). Blood was collected from jugular vein using 16G catheter into acid citrate dextrose A (ACD-A) coated syringes. Complete blood count was performed by Advia hematology systems.

**Fig. 1:**
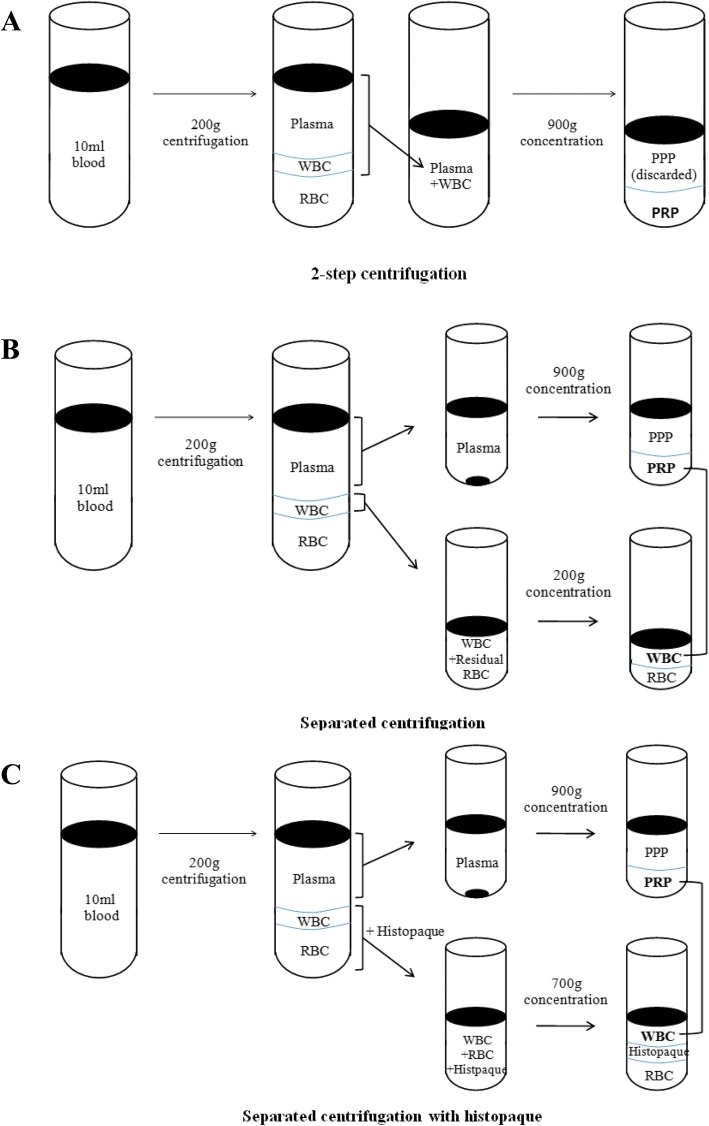
Summary of the methods for platelet rich plasma preparation. **a**, 2-step centrifugation. **b** Separated centrifugation. **c** Separated centrifugation with histopaque

In a 2-step centrifugation method, blood was centrifuged at 200 g for 15 min. A layer of platelet rich plasma and white blood cells were collected. The suspension was centrifuged again at 900 g for 15 min. The supernatant was discarded and 1.5 ml of platelet rich plasma was collected and analyzed by Advia hematology systems. In a separated centrifugation method, blood was centrifuged at 200 g for 15 min and plasma was collected. Residual white blood cells and red blood cells were collected in a different tube. Plasma was centrifuged at 900 g for 15 min, the supernatant was discarded and 1 ml of plasma was collected. Residual blood cells were centrifuged at 200 g for 15 min and the supernatant was collected. Collected plasma and white blood cells were mixed together, then complete blood was counted. In a separated centrifugation using histopaque (Histopaque® -1119, Sigma, St. Louis, USA) method, blood was centrifuged at 200 g for 15 min and plasma was collected and then centrifuged again at 900 g for 15 min. The supernatant was discarded and collected the plasma. Residual blood cells were collected into a different tube and mixed with PBS (1:1). Histopaque was added into the tube and the suspension was centrifuged at 400 g for 30 min. White blood cells were separated and washed with PBS, then centrifuged again at 200 g for 10 min. PBS was removed and white blood cells were added into the plasma tube. Complete blood count was performed (Fig. 1). All the data obtained was analyzed by a statistical software (SPSS Inc., IBM, USA) using non-parametric Mann-Whitney (statistical significance was considered at *P* < 0.05) and Kruskal-Wallis tests (statistical significance was considered at *P* < 0.017).

## Results

Red blood cells were significantly lower in the method of separated centrifugation using histopaque (mean 0.09 ± 0.05 × 10^6^/ ul) and significantly higher in the 2-step centrifugation method (mean 8.50 ± 2.31 × 10^6^/ ul) than in other groups. The significantly higher number of white blood cells was shown in the 2-step centrifugation (mean 35.20 ± 3.65 × 10^3^/ ul) and the lowest number was shown with separated centrifugation with histopaque method (mean 12.53 ± 2.59 × 10^3^/ ul). However, a significant difference of the number of white blood cells between the separated centrifugation and separated centrifugation with histopaque was not found. The platelets were the most concentrated with separated centrifugation with histopaque method. In the 2-step centrifugation, separated centrifugation and separated centrifugation with histopaque, platelets were concentrated 4.5, 5.3 and 5.6 times, respectively. However, no significant difference of the platelet concentration between the three groups was found (Table 1).

**Table 1:** Results of platelet rich plasma analysis

Horse		RBC				WBC				Platelet			
		W^b,c^	A^b,c^	B^w,a,b^	C^w,a,b,c^	W^a,b,c^	A^w,b,c^	B^w,a^	C^w,a^	W^a,b,c^	A^w^	B^w^	C^w^
1	Halla Female 13 yrs	6.96	9.03	2.51	0.14	7.04	36.56	14.85	17.06	79	368	556	605
2	Halla Male 7 yrs	7.07	10.82	4.15	0.11	5.94	33.25	18.55	10.69	71	274	439	380
3	TB Female 4 yrs	11.26	11.56	4.65	0.12	8.88	34.77	19.2	13.5	104	503	680	650
4	TB Female 4 yrs	7.76	6.45	2.46	0.03	7.8	34.1	21.3	13	106	602	427	629
5	Halla Male 7 yrs	6.92	6.57	2	0.02	7.9	41.6	12	10.7	133	494	543	499
6	Halla Female 13 yrs	6.61	6.57	4.56	0.13	7.97	30.89	22.72	10.25	118	497	610	652
Mean		7.76	8.50	3.39	0.09	7.59	35.20	18.10	12.53	101.83	456.33	542.50	569.17
SD		1.75	2.31	1.19	0.05	1.00	3.65	4.02	2.59	23.35	116.20	97.67	108.60

*A* 2-setp centrifugation, *B* Separated centrifugation, *C* Separated centrifugation with histopaque, *TB* Thoroughbred, *W* Whole blood, *RBC* Red blood cell, *WBC* White blood cell, ^w,a,b,c^Different letters represent significant differences between groups (*P*-value: W vs. A, B, C, Mann-Whitney test, *P* < 0.05; A vs. B vs. C, Kruskal-Wallis test, *P* < 0.017)

## Discussion

In a recent study, platelet rich plasma has been thought to take a therapeutic effect in the aspect of not only platelets but also red blood cells and white blood cells [9]. Red blood cells and white blood cells have been identified to have important roles in immune-mediated response [9]. Therefore, regulating the amount of blood cells was important in this study. Since red blood cells increased immune-mediated factors such as interleukin-1 and TGF-α, it is important to reduce the amount of red blood cells during preparation of platelet rich plasma [6]. Also, it was demonstrated that immune-mediated factors were increased when there were high concentration of red blood cells in platelet rich plasma which have the low number of white blood cells [6]. The efforts to reduce red blood cells were conducted by using single or double centrifugation [10] and difference of time and the gravitational force of centrifugation [11]. In this study, separated centrifugation with histopaque showed the significantly lower number of red blood cells than the 2-step centrifugation and separated centrifugation. This is because histopaque separated the layer of white blood cells from the layer of red blood cells.

The necessity of white blood cells in platelet rich plasma is still controversial. McCarrel T et al. observed that white blood cells and corticosteroids were effective in the treatment of chronic lateral epicondylitis in horse [12]. Also, platelet rich plasma, including white blood cells except neutrophils had an effect on anterior cruciate ligament fibroblast [13]. Therefore, we had effort to retain white blood cells in platelet rich plasma. However, there are some adverse effects when white blood cells are concentrated [12]. Immune-mediated factors such as interlukin-1 and TGF-α could be increased because of concentrated neutrophils [11] and platelet rich plasma, including a high concentration of white blood cells decreased synthesis of extracellular matrix [9]. In this study, white blood cells were significantly higher in the 2-step centrifugation method than other groups. The lowest number of white blood cells was observed with the method of separated centrifugation using histropaque but not significantly different with the separated centrifugation. This is may be due to loss of white blood cells during washing procedure.

Different methods for preparation of platelet rich plasma has been developed [6, 7, 9, 13]. The proper number of platelets of platelet rich plasma was three to five times higher than whole blood [6]. We prepared platelet rich plasma with the proper number of platelets, since the concentration of platelets in 2-step centrifugation, separated centrifugation and separated centrifugation with histopaque method was 4.5, 5.3 and 5.6 times respectively, with no significant difference.

## Conclusion

The purpose of this study was comparing the methods for platelet rich plasma preparation to ascertain the most appropriate method. We intended to reduce red blood cells and to preserve white blood cells during the process of platelet rich plasma. The method of using histopaque was considered the most appropriate since platelets were concentrated while red blood cells were removed the most and white blood cells were included.
